# Supplementary material for: Quantitative 3D imaging of the cranial microvascular environment at single-cell resolution
Source: Nat Commun. 2021 Oct 28;12:6219. doi: 10.1038/s41467-021-26455-w (PMC8553857; doi:10.1038/s41467-021-26455-w)
Supplement: Supplementary file 1 — Supplementary Information [file 41467_2021_26455_MOESM1_ESM.pdf]

Supplementary Information

Quantitative 3D Imaging of the Cranial Microvascular Environment at Single-Cell Resolution

Alexandra N. Rindone, Xiaonan Liu, Stephanie Farhat, Alexander Perdomo-Pantoja, Timothy F. Witham, Daniel L. Coutu, Mei Wan, Warren L. Grayson

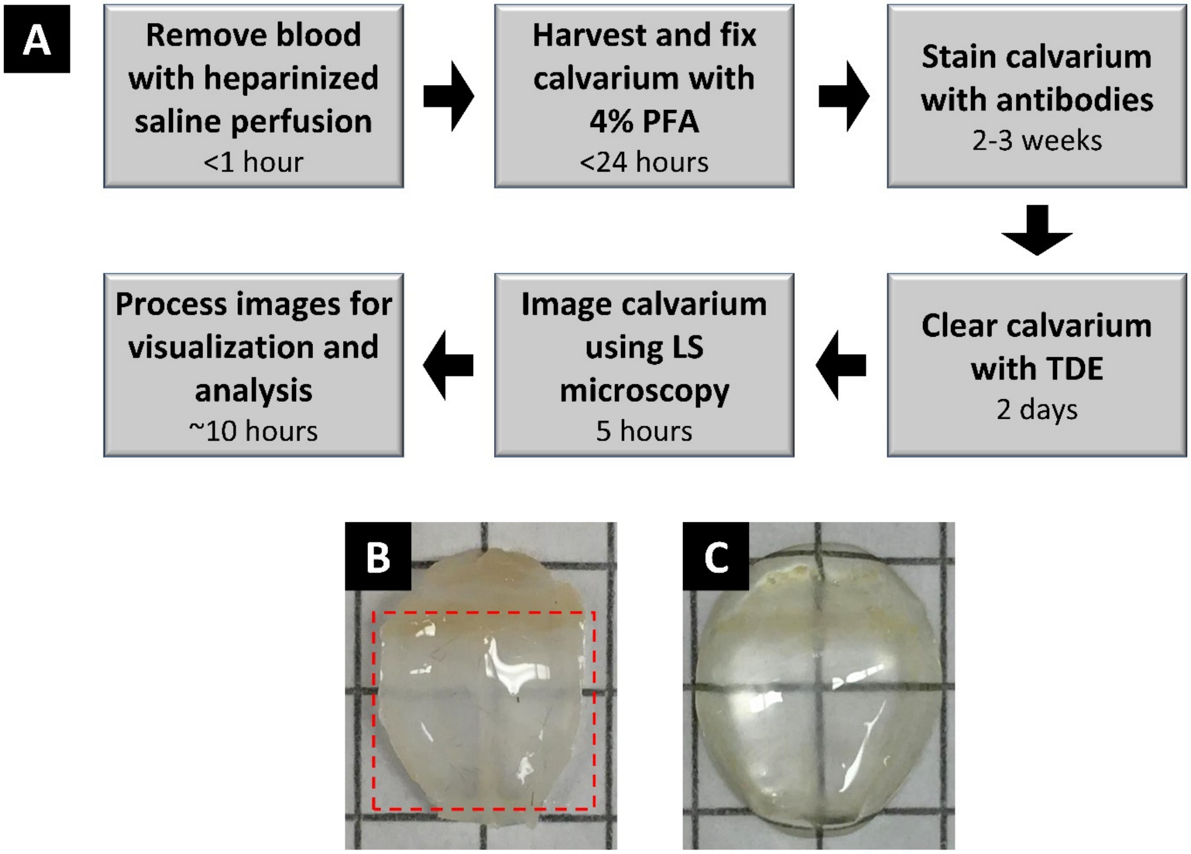

Supplemental Figure 1. Quantitative 3D light-sheet imaging pipeline. A) Diagram displaying the steps to harvest, stain, clear, and image calvaria for data analysis. The entire procedure—including quantitative analysis—takes 3-4 weeks to complete. B) Non-decalcified calvarium before optical clearing. The dotted red lines indicate the region of the calvarium that was imaged. C) Non-decalcified calvarium following clearing with TDE.

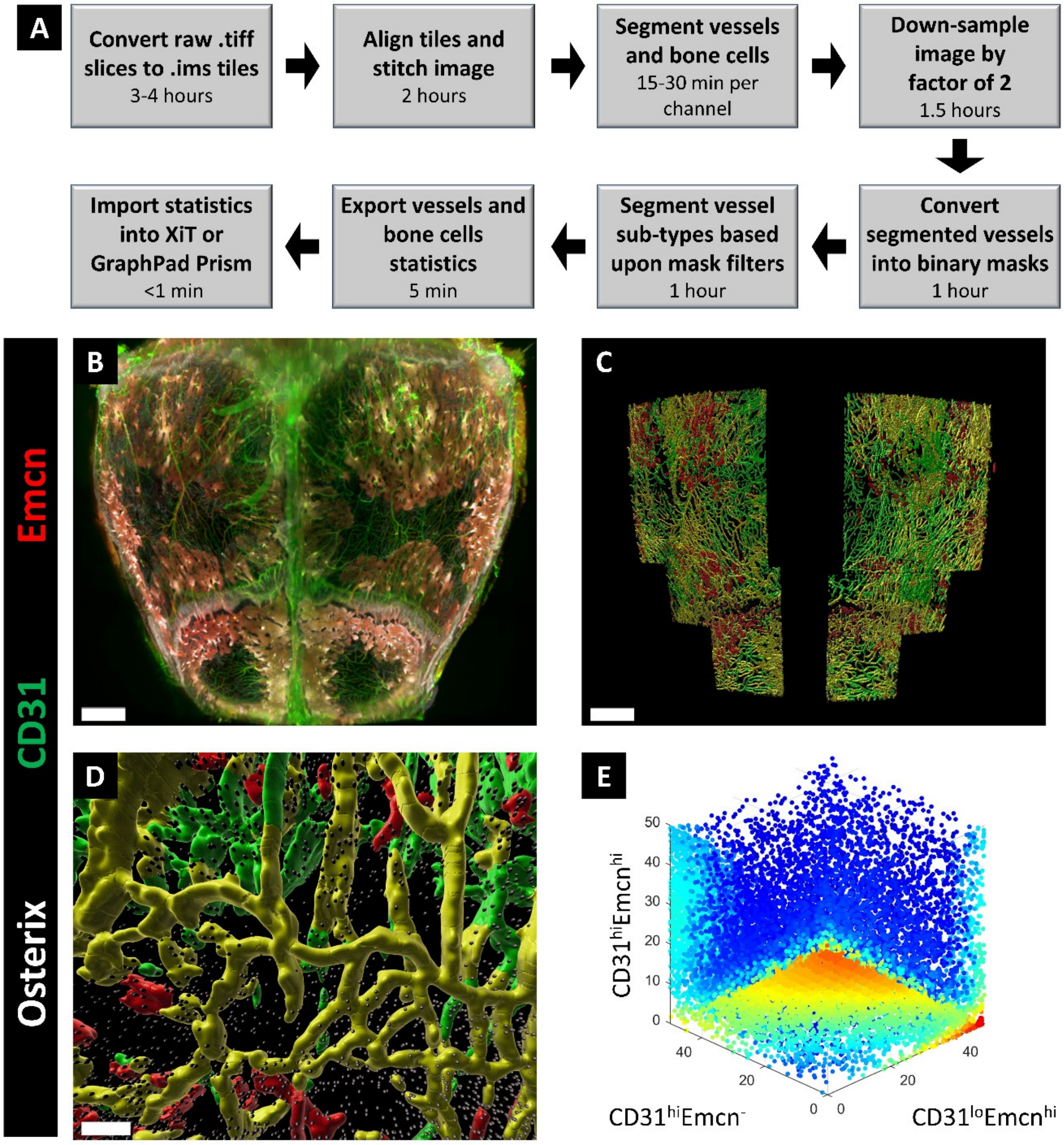

Supplemental Figure 2. Quantitative analysis pipeline for characterizing calvarial blood vessel phenotype and skeletal progenitor distribution in 3D. A) Diagram displaying the steps performed to analyze each dataset. The entire pipeline takes approximately 10 hours to complete using a high-performance workstation. B) 3D projection of a calvarium stained for CD31, Emcn, and Osterix. C) 3D segmentation of calvarial vessel phenotypes and Osterix+ cells in the volume of interest (sagittal suture not included due to the interference of signal from the sagittal sinus). D) Zoomed-in portion of (C) displaying the spatial relationships of Osterix+ cells to each vessel phenotype. Segmented colors represent the following: $CD31^{hi}Emcn^{-}$ vessels (green), $CD31^{hi}Emcn^{hi}$ vessels (yellow), $CD31^{lo}Emcn^{hi}$ vessels (red), Osterix+ cells (gray). Segmentation results are representative of the 32 whole calvaria imaged and analyzed in this study (different regions of interest were used to analyze the 12 calvaria with subcritical-sized defects). E) 3D plot representing the distance of individual Osterix+ cells to each vessel phenotype from (C). Dot color represents the density of cells relative to their position in the plot (red=high density, blue=low density). Scale bars: 1000 µm (B,C); 200 µm (D). Colors (B-D): Red: Endomucin (Emcn), Gray: Osterix, Green: CD31

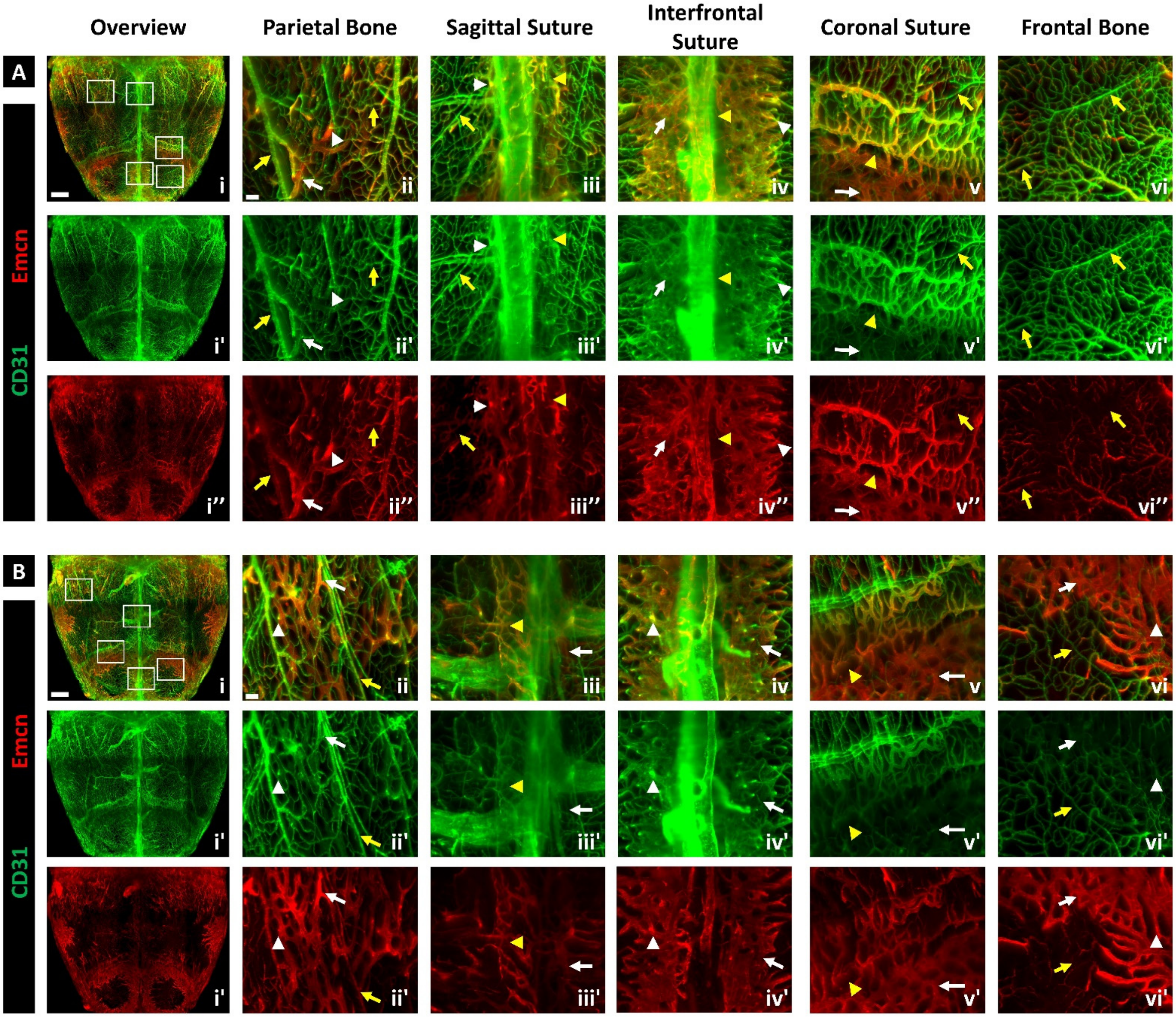

Supplemental Figure 3. 3D map of vessel phenotypes in the calvaria of 4-week-old mice. A-B) MIP of vessel phenotypes in two independent calvaria, as originally depicted in Figure 2. Aii-vi and Bii-vi represent different regions of the calvaria marked in Ai and Bi. CD31^{hi}Emcn^{-} and CD31^{hi}Emcn^{hi} vessels in the periosteum and dura mater (yellow arrows) connect to CD31^{hi}Emcn^{hi} and CD31^{lo}Emcn^{hi} marrow vessels via CD31^{hi}Emcn^{hi} transcortical vessels (white arrowheads). Vessels in the periosteum are prevalent across the parietal and frontal bones, while marrow vessels are restricted to regions nearby the sutures (yellow arrowheads). Results were replicated in 7 calvaria from 4-week-old mice (images from 2 calvaria are shown). Scale bars: 1000 μm (Ai, Bi), 100 μm (Aii-v, Bii-v). Colors: Red: Endomucin (Emcn), Green: CD31

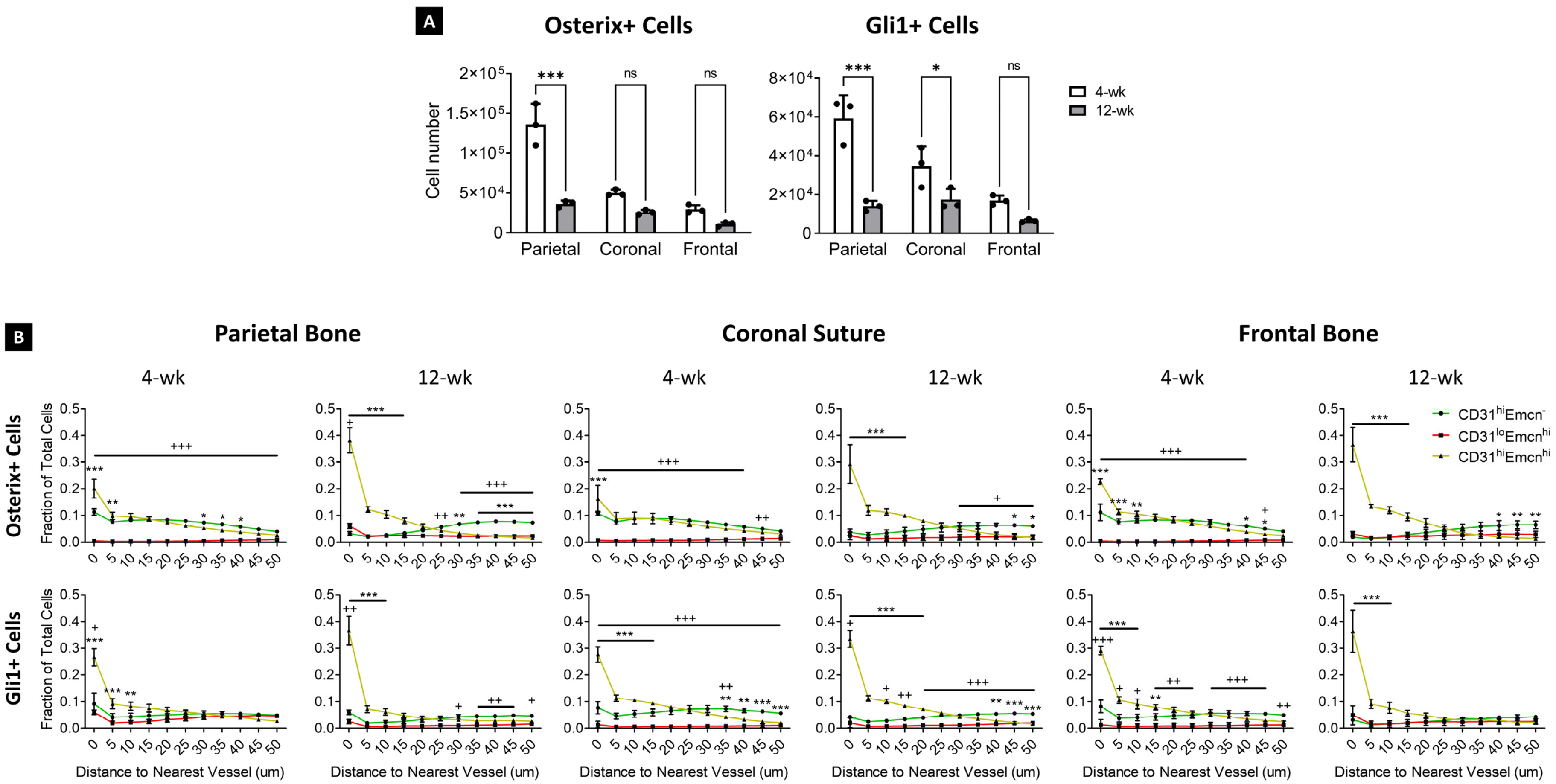

Supplemental Figure 4. Distribution of skeletal progenitors in different regions of the calvarium. A) Number of Osterix+ and Gli1+ cells in each region for 4-wk and 12-wk-old mice (n=3). B) Plots showing the nearest distance of Osterix+ (top) and Gli1+ (bottom) progenitors to each vessel phenotype (n=3) in the parietal bones, frontal bones, and coronal suture (including parietal/frontal bones just adjacent to the suture). Data are mean ± SD. Statistics were performed using a two-way ANOVA and Bonferroni post-hoc test. ***p<0.001, **p<0.01, *p<0.05 where designated or between $CD31^{hi}Emcn^{hi}$ and $CD31^{hi}Emcn^{-}$ vessels; +++p<0.001, ++p<0.01, +p<0.05 between $CD31^{lo}Emcn^{hi}$ and $CD31^{hi}Emcn^{-}$ vessels

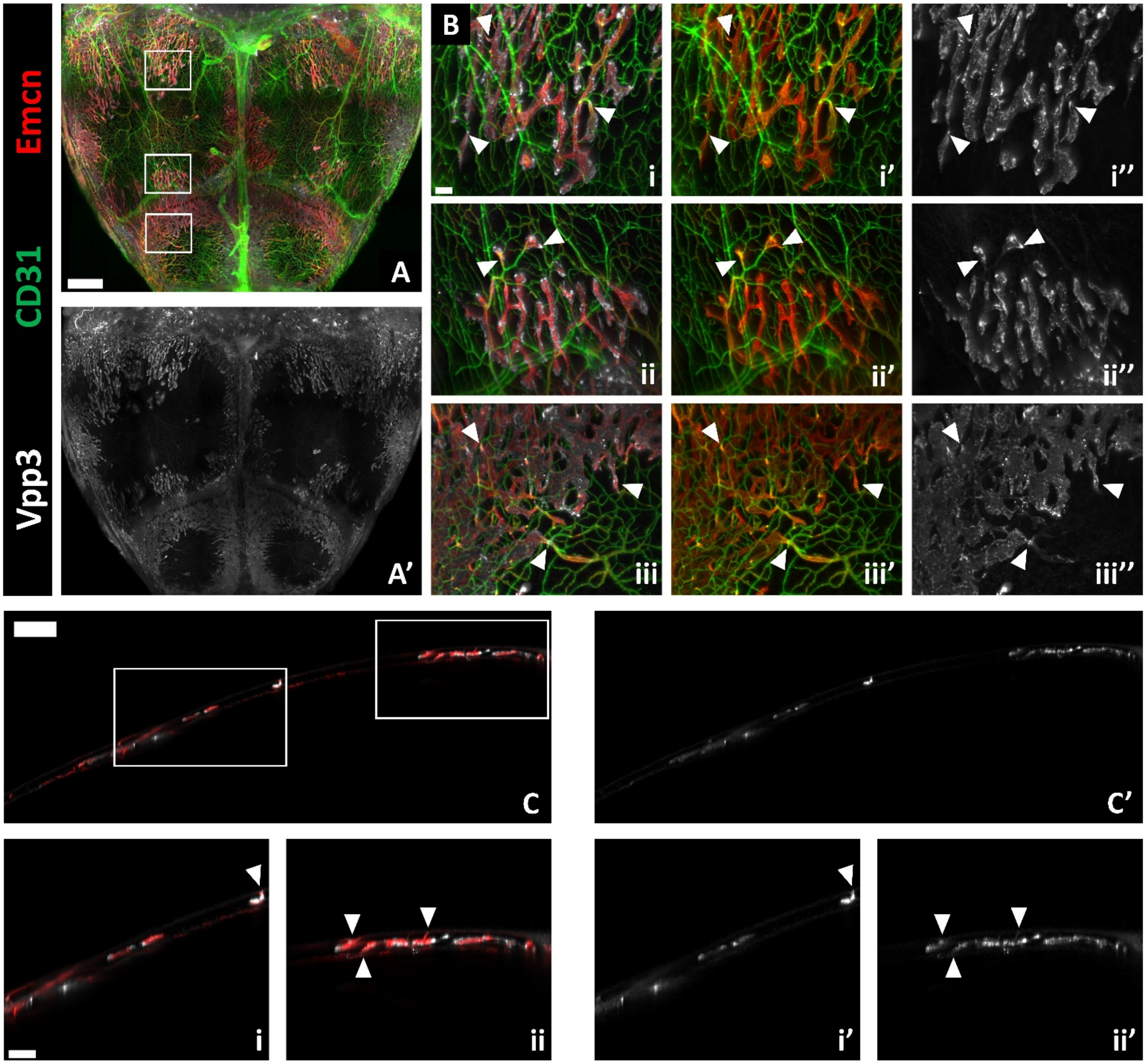

Supplemental Figure 5. Osteoclasts reside in the marrow cavities and transcortical canals. A) MIP of vessels and Vpp3+ osteoclasts in the calvarium from a 4-week-old mouse. B) Zoomed-in regions from (A) demonstrating the spatial distribution of osteoclasts relative to vessels. Arrowheads point to regions where osteoclasts reside at or adjacent to transcortical canals. C) 40 µm-thick sagittal section displaying the distribution of osteoclasts relative to $Emcn^{hi}$ transcortical and marrow vessels. Arrowheads indicate regions where osteoclasts reside nearby $Emcn^{hi}$ transcortical vessels. Images were acquired from 1 calvarium. Scale bars: 1000 µm (A); 500 µm (C); 200 µm (Ci-ii); 100 µm (B). Colors: Red: Endomucin (Emcn), Gray: Vpp3, Green: CD31

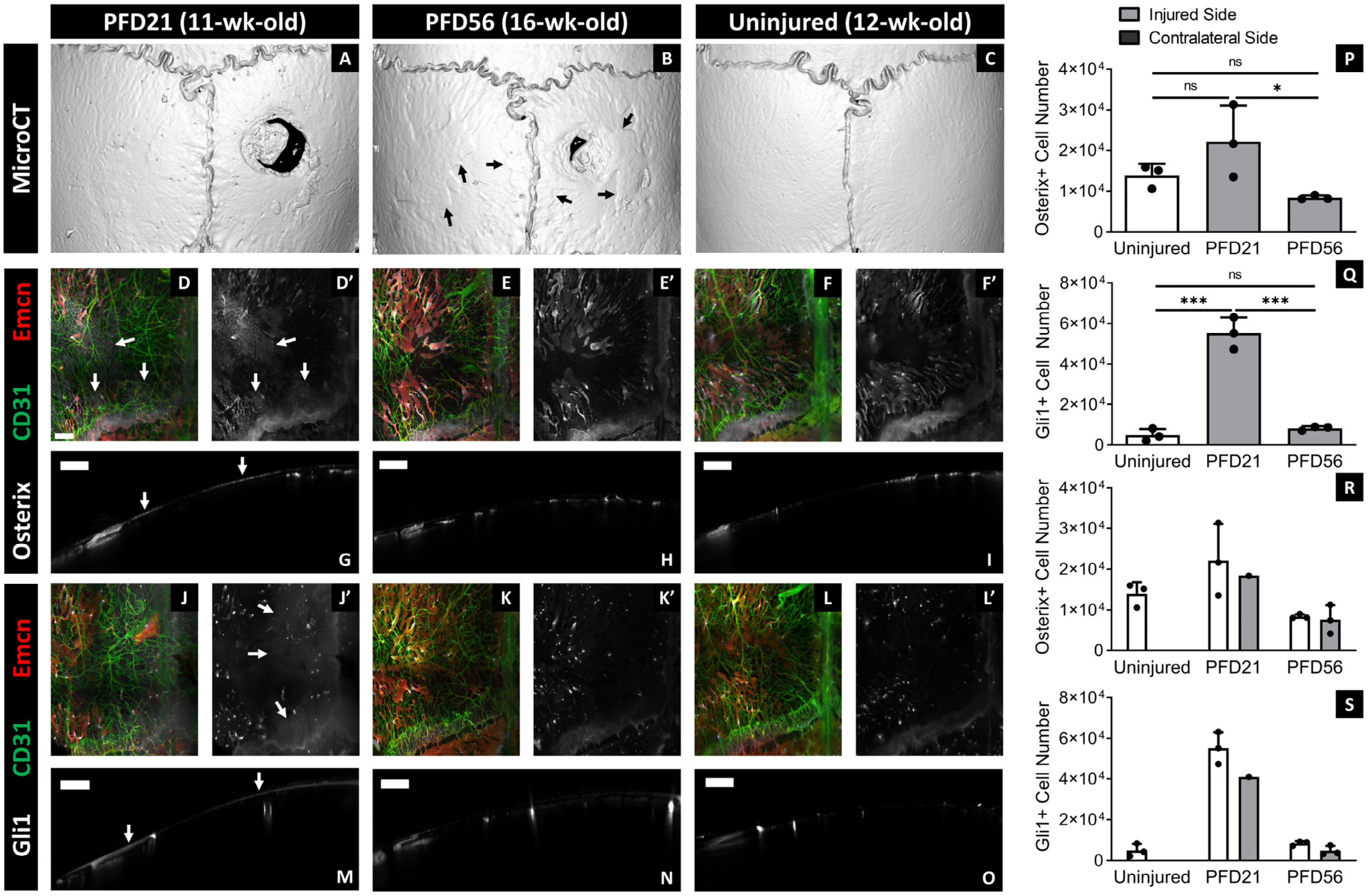

Supplemental Figure 6. Localized calvarial injury stimulates systemic skeletal progenitor expansion. A-C) MicroCT 3D surface renderings of injured calvaria at PFD21 (A) and PFD56 (B) compared to an uninjured calvarium (C). Black arrows point to regions of excess mineral deposition along the cortical surface of injured calvaria (B). Results were replicated in 3 calvaria from the 11- and 12-wk-old mice and 4 calvaria from the 16-wk-old mice. D-F) MIP of calvarial vessels and Osterix+ skeletal progenitors in the contralateral parietal bone at PFD21 (D) and PFD56 (E) compared to an uninjured control (F). G-I) 40 µm-thick sagittal sections from (D-F) displaying distribution of Osterix+ progenitors along the thickness of the contralateral parietal bone. J-L) MIP of calvarial vessels and Gli1+ skeletal progenitors in the contralateral parietal bone at PFD21 (J) and PFD56 (K) compared to an uninjured control (L). M-O) 40 µm-thick sagittal sections from (J-K) displaying distribution of Gli1+ progenitors along the thickness of the contralateral parietal bone. White arrows mark regions of skeletal progenitor expansion within the periosteum at PFD21 (D, G, J, M). Images were acquired in 1 calvarium for each experimental group (D-O). P-Q) Quantification of skeletal progenitors in the ipsilateral side of the injured parietal bone compared to the uninjured control (n=3). R-S) Quantification of skeletal progenitors in the ipsilateral and contralateral sides of the injured parietal bone compared to the uninjured control (n=3 for control, ipsilateral PFD21, ipsilateral and contralateral PFD56; n=1 for contralateral PFD21). Data are mean ± SD. Statistics were performed using a one-way ANOVA with Tukey post-hoc test (P, Q). ***$p<0.001$, *$p<0.05$ where designated. Scale bars: 500 µm (D-O). Colors: Red: Endomucin (Emcn), Gray: Osterix (D-I) or Gli1 (J-O), Green: CD31

Supplemental Table 1. List of all key reagents and resources used in this study.

Reagent or resource	Source	Identifier
Antibodies		
Goat anti-mouse/rat CD31 (1:100)	R&D Systems	AF3628
Rat anti-mouse/rat Endomucin (1:50)	Santa Cruz Biotechnology	sc-65495
Rabbit anti-mouse/rat/human Osterix (1:200)	Abcam	ab209484
Rabbit anti-mouse/human Gli1 (1:100)	Sigma Aldrich	SAB4301901-100UL
Rabbit anti-mouse/rat/human Vpp3 (ATP6V1B1 + ATP6V1B2; 1:200)	Abcam	ab200839
Donkey anti-goat AF800 plus, 0.67 mg/mL, (1:50)	Thermo Fisher Scientific	A32930
Donkey anti-rabbit AF647 plus, 0.67 mg/mL (1:150)	Thermo Fisher Scientific	A32795
Donkey anti-rat biotin, 0.75 mg/mL (1:100)	Thermo Fisher Scientific	A18749
Streptavidin AF555 conjugate, 0.67 mg/mL (1:100)	Thermo Fisher Scientific	S32355
Reagents		
Heparin sodium salt from porcine mucosa	Sigma Aldrich	H3393-50KU
Paraformaldehyde, 16% aq. soln., methanol free	Alfa Aesar	433689M
Normal donkey serum	Sigma Aldrich	D9663-10ML
Trizma base	Sigma Aldrich	T6066-1KG
Trizma hydrochloride	Sigma Aldrich	T5941-1KG
Sodium chloride	Sigma Aldrich	S5886
Tween 20	Sigma Aldrich	P7949
Dimethylsulfoxide	Thermo Fisher Scientific	PI20688
Streptavidin/Biotin Blocking Kit	Vector Laboratories	SP-2002
2,2-thiodiethanol	Sigma Aldrich	166782-500G
Animal drugs and materials		
Ketamine HCl (100 mg/mL)	VetOne	501072
Xylazine Injection (20 mg/mL)	Akorn, Inc.	N/A

Reagent or resource	Source	Identifier
pTH (1-34)	Bachem	4011474
Povidone-Iodine Swabsticks	Fisher Scientific	06-669-83
6-0 nylon monofilament sutures	Ethicon	1665G
Buprenorphine SR-LAB (0.5 mg/mL)	ZooPharm	N/A
Ideal Micro-Drill	Harvard Apparatus	72-6065
1 mm carbide inverted cone burr	Roboz Surgical Instrument Co.	RS-6282C-35
Stereotactic frame	KOPF, David Kopf Instruments	900LS
Surgical microscope	Carl Zeiss	NC-4
Instruments and hardware		
Ultramicroscope II, 2x zoom body configuration	Miltenyi Biotec (formerly manufactured by LaVision Biotec)	N/A
LVMI-Fluor 2x objective lens with dipping cap (0.5 NA, 1.33-1.57 RI range, 5.6 mm WD)	Miltenyi Biotec (formerly manufactured by LaVision Biotec)	N/A
Dell Precision 7820 Tower	Dell	T7820X
Portable SSD T5 USB 3.1 2 TB	Samsung	MU-PA2T0B
Skyscan 1275 µCT	Bruker	N/A
Software		
Imaris 9.5 Single Full with ClearView plus Stitcher	Bitplane Inc.	N/A
XiT	See ref. [14]	N/A
CTAn, CTVol, and CTVox	Bruker	N/A
Mimics 14	Materialise	N/A
Microsoft Excel 2019	Microsoft	N/A
GraphPad Prism 5	GraphPad Software	N/A
