# Supplementary material for: Quantitative 3D imaging of the cranial microvascular environment at single-cell resolution
Source: Nat Commun. 2021 Oct 28;12:6219. doi: 10.1038/s41467-021-26455-w (PMC8553857; doi:10.1038/s41467-021-26455-w)
Supplement: Supplementary file 3 — Description of Additional Supplementary Files [file 41467_2021_26455_MOESM3_ESM.docx]

Description of Additional Supplementary Files

File Name: Supplementary Video 1

Description: 3D projection of blood vessels and Osterix+ skeletal progenitors in the murine calvarium. Fluorescent labels are denoted using the following pseudo colors: green (CD31), red (Endomucin), and gray (Osterix). Segmentation is shown for the following: CD31^hi^Emcn^-^ arteries and arterioles (green), CD31^hi^Emcn^hi^ capillaries (gold), CD31^lo^Emcn^hi^ capillaries and sinusoids (red), and Osterix+ skeletal progenitors (gray).
