# Supplementary material for: Quantitative 3D imaging of the cranial microvascular environment at single-cell resolution
Source: Nat Commun. 2021 Oct 28;12:6219. doi: 10.1038/s41467-021-26455-w (PMC8553857; doi:10.1038/s41467-021-26455-w)
Supplement: Supplementary file 5 — Reporting Summary [file 41467_2021_26455_MOESM5_ESM.pdf]

nature research

Corresponding author(s): Warren L. Grayson

Last updated by author(s): 9/21/2021

Reporting Summary

Nature Research wishes to improve the reproducibility of the work that we publish. This form provides structure for consistency and transparency in reporting. For further information on Nature Research policies, see our Editorial Policies and the Editorial Policy Checklist.

Statistics

For all statistical analyses, confirm that the following items are present in the figure legend, table legend, main text, or Methods section.

n/a	Confirmed	
☐	☒	The exact sample size (*n*) for each experimental group/condition, given as a discrete number and unit of measurement
☐	☒	A statement on whether measurements were taken from distinct samples or whether the same sample was measured repeatedly
☐	☒	The statistical test(s) used AND whether they are one- or two-sided<br>*Only common tests should be described solely by name; describe more complex techniques in the Methods section.*
☒	☐	A description of all covariates tested
☒	☐	A description of any assumptions or corrections, such as tests of normality and adjustment for multiple comparisons
☐	☒	A full description of the statistical parameters including central tendency (e.g. means) or other basic estimates (e.g. regression coefficient) AND variation (e.g. standard deviation) or associated estimates of uncertainty (e.g. confidence intervals)
☐	☒	For null hypothesis testing, the test statistic (e.g. *F*, *t*, *r*) with confidence intervals, effect sizes, degrees of freedom and *P* value noted<br>*Give P values as exact values whenever suitable.*
☒	☐	For Bayesian analysis, information on the choice of priors and Markov chain Monte Carlo settings
☒	☐	For hierarchical and complex designs, identification of the appropriate level for tests and full reporting of outcomes
☒	☐	Estimates of effect sizes (e.g. Cohen's *d*, Pearson's *r*), indicating how they were calculated

Our web collection on statistics for biologists contains articles on many of the points above.

Software and code

Policy information about availability of computer code

Data collection	ImSpector Pro 5.1.328 (LaVision BioTec; now owned by Miltenyi Biotec) used for light-sheet image acquisition
Data analysis	Imaris 9.5 for Core Facilities (Bitplane) and XiT Software v1 (see ref. 14) used for light-sheet image visualization, processing, and analysis<br>Mimics 14, CTAn 1.20.3.0, CTVol 2.3.2.0, CTVox 3.3.1 used for microCT image visualization, processing, and analysis<br>GraphPad Prism 5 and Microsoft Excel 2019 used for data analysis and statistics

For manuscripts utilizing custom algorithms or software that are central to the research but not yet described in published literature, software must be made available to editors and reviewers. We strongly encourage code deposition in a community repository (e.g. GitHub). See the Nature Research guidelines for submitting code & software for further information.

Data

Policy information about availability of data

All manuscripts must include a data availability statement. This statement should provide the following information, where applicable:

- Accession codes, unique identifiers, or web links for publicly available datasets
- A list of figures that have associated raw data
- A description of any restrictions on data availability

The data supporting the findings from this study are available within the article file and its supplementary information. Source data are provided with this paper. The 3D microscopy data generated in this study have been deposited in the BioImage Archive database under accession code S-BIAD171 [https://www.ebi.ac.uk/biostudies/BioImages/studies/S-BIAD171]. Any remaining raw data will be available from the corresponding author upon reasonable request.

Field-specific reporting

Please select the one below that is the best fit for your research. If you are not sure, read the appropriate sections before making your selection.

- [x] Life sciences
- [ ] Behavioural & social sciences
- [ ] Ecological, evolutionary & environmental sciences

For a reference copy of the document with all sections, see nature.com/documents/nr-reporting-summary-flat.pdf

Life sciences study design

All studies must disclose on these points even when the disclosure is negative.

Sample size	No statistical methods were used to pre-determine sample size prior to the study. For light-sheet image analysis, all experiments used at least 3 biological replicates to allow for statistical analysis to be formed using a two-tailed t-test, one-way ANOVA with Tukey's post-hoc test, or two-way ANOVA with Bonferroni's post-hoc test. We found this to be sufficient to detect statistical differences between groups.<br><br>For microCT imaging and analysis, all experiments used at least 4 biological replicates, and our sample size based on prior experiments (calvarial defects: https://doi.org/10.1089/ten.tea.2018.0341; Trap-Cre PDGFfl/fl mice: https://doi.org/10.1172/JCI98857) and literature (all other groups: https://doi.org/10.3389/fphys.2017.00697).
Data exclusions	No data was excluded from analysis.
Replication	All experiments were performed using at least three biological replicates (i.e. calvaria from at least 3 mice). All attempts at replication were successful.<br><br>Methods for staining, clearing, and light-sheet imaging were repeated with several samples (4 whole calvaria, 10-15 half calvaria) prior to performing the experiments presented in this manuscript. All replication attempts were successful.
Randomization	All experiments were randomized.
Blinding	Blinding was not performed for light-sheet image acquisition and data analysis. We used the same settings to image all samples, and therefore blinding was not necessary for light-sheet image acquisition. Methods for performing light-sheet image analysis were semi-automated, and involved setting thresholds that varied between different experiments (due to differences in maximum signal intensity between some groups with higher bone thickness, such as 12-week-old mice compared to 4-week-old mice). With our semi-automated pipeline, we expect that consistent results would be obtained between different experiments and researchers.<br><br>Blinding was performed for microCT image acquisition. Blinding was not performed for image analysis because the segmentation was automated and consistent across all samples and groups (described in detail in the Methods).

Reporting for specific materials, systems and methods

We require information from authors about some types of materials, experimental systems and methods used in many studies. Here, indicate whether each material, system or method listed is relevant to your study. If you are not sure if a list item applies to your research, read the appropriate section before selecting a response.

Materials & experimental systems

n/a	Involved in the study	
[ ]	[x]	Antibodies
[x]	[ ]	Eukaryotic cell lines
[x]	[ ]	Palaeontology and archaeology
[ ]	[x]	Animals and other organisms
[x]	[ ]	Human research participants
[x]	[ ]	Clinical data
[x]	[ ]	Dual use research of concern

Methods

n/a	Involved in the study	
[x]	[ ]	ChIP-seq
[x]	[ ]	Flow cytometry
[x]	[ ]	MRI-based neuroimaging

Antibodies

Antibodies used	Goat anti-mouse/rat CD31 (1:100) R&D Systems AF3628<br>Rat anti-mouse/rat Endomucin (1:50) Santa Cruz Biotechnology sc-65495<br>Rabbit anti-mouse/rat/human Osterix (1:200) Abcam ab209484<br>Rabbit anti-mouse/human Gli1 (1:100) Sigma Aldrich SAB4301901-100UL<br>Rabbit anti-mouse/rat/human Vpp3 (ATP6V1B1 + ATP6V1B2; 1:200) Abcam ab200839<br>Donkey anti-goat AF800 plus, 0.67 mg/mL, (1:50) Thermo Fisher Scientific A32930<br>Donkey anti-rabbit AF647 plus, 0.67 mg/mL (1:150) Thermo Fisher Scientific A32795

	Donkey anti-rat biotin, 0.75 mg/mL (1:100) Thermo Fisher Scientific A18749<br>Streptavidin AF555 conjugate, 0.67 mg/mL (1:100) Thermo Fisher Scientific S32355
Validation	The following primary antibodies were validated from published studies using similar sample types and fixation methods:<br>Goat anti-mouse/rat CD31, rat anti-mouse/rat Endomucin: Coutu, D., Kokkaliaris, K., Kunz, L. et al. Multicolor quantitative confocal imaging cytometry. Nat Methods 15, 39–46 (2018). https://doi.org/10.1038/nmeth.4503<br><br>Rabbit anti-mouse/rat/human Vpp3 (ATP6V1B1 + ATP6V1B2): Romeo, S.G., Alawi, K.M., Rodrigues, J. et al. Endothelial proteolytic activity and interaction with non-resorbing osteoclasts mediate bone elongation. Nat Cell Biol 21, 430–441 (2019). https://doi.org/10.1038/s41556-019-0304-7<br><br>Rabbit anti-mouse/rat/human Osterix was validated for immunohistochemistry (paraffin-embedded) using a dilution of 1:1000 and Tris/EDTA-mediated antigen retrieval by the manufacturer (Abcam, ab209484). Rabbit anti-mouse/human Gli1 was validated for immunohistochemistry (paraffin-embedded) using a 1:20-1:100 dilution by the manufacturer (Sigma Aldrich, SAB4301901).<br>We independently validated these antibodies by comparing the location and appearance of each stain to findings from other published studies (Zhao H, Feng J, Ho TV, et al., The suture provides a niche for mesenchymal stem cells of craniofacial bones. Nat Cell Biol 17, 386-396 (2015). https://doi.org/10.1038/ncb3139; Sivaraj KK, Jeong HW, Dharmalignam B, et al., Regional specialization and fate specification of bone stromal cells in skeletal development. Cell Rep 36, 109352 (2021). https://doi.org/10.1016/j.celrep.2021.109352).

Animals and other organisms

Policy information about studies involving animals; ARRIVE guidelines recommended for reporting animal research

Laboratory animals	We purchased the following mouse strains from Jackson Laboratories: C57BL/6J (Stock No. 000664) and Pdgfbfl/fl (Stock No. 017622). All C57BL/6J mice used in this study were male, and were either 4-weeks, 8-weeks, or 12-weeks of age (ages for specific experiments specified in the Methods).<br><br>We obtained Trap-cre mice from J.J. Windle (Virginia Commonwealth University, Richmond, VA, USA). We crossed hemizygous Trap-cre mice with Pdgfbfl/fl mice to produce Trap-cre Pdgfbfl/fl offspring (referred to as PdgfbcKO in the Main text, Pdgfbfl/fl littermates referred to as "WT"). WT and PdfgbcKO mice used for the experiments were female and 4-weeks-old.<br><br>Mice were housed and cared for in Johns Hopkins Research Animal Resources facilities in rooms with controlled temperature and a standard 12:12 hour light-dark cycle. Mice were provided food and water ad libitum.
Wild animals	No wild animals were used in this study.
Field-collected samples	No field-collected samples were used in this study.
Ethics oversight	All animal experiments were approved by the Johns Hopkins University Institutional Animal Care and Use Committee (Protocol No. MO18M188).

Note that full information on the approval of the study protocol must also be provided in the manuscript.
